# Supplementary material for: The interactome of intact mitochondria by cross-linking mass spectrometry provides evidence for coexisting respiratory supercomplexes
Source: Mol Cell Proteomics. 2017 Dec 8;17(2):216–32. doi: 10.1074/mcp.RA117.000470 (PMC5795388; doi:10.1074/mcp.RA117.000470)
Supplement: Supplemental Data [file supp_RA117.000470_133922_0_supp_23978_fzffwf.zip › spectra_annotation/mito_DR_spectra_annotation/430-1-10-1-8-1.pdf]

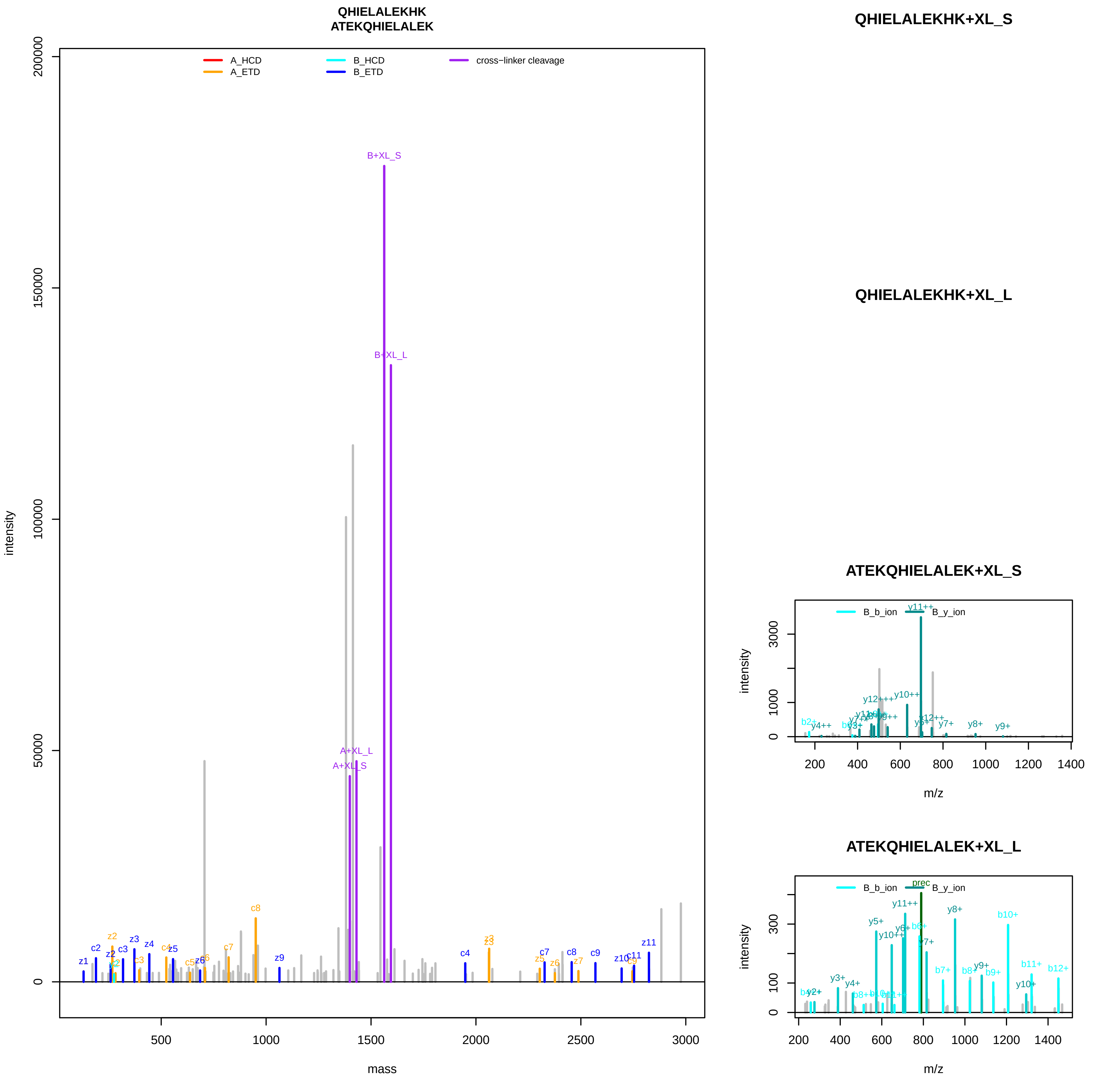
QHIELALEKHK
ATEKQHIELALEK
A_HCD
A_ETD
B_HCD
B_ETD
cross−linker cleavage
B+XL_S
B+XL_L
A+XL_L
A+XL_S
intensity
mass
QHIELALEKHK+XL_S
QHIELALEKHK+XL_L
ATEKQHIELALEK+XL_S
B_b_ion
B_y_ion
y11++
y10++
y12+++
y7++
y8+
y9+
m/z
ATEKQHIELALEK+XL_L
prec
y11++
y8+
b10+
y5+
y10++
b6+
b7+
b8+
b9+
b11+
b12+
y10+
y3+
y4+
